# Single-cell RNA sequencing reveals distinct tumor microenvironment of ground glass nodules and solid nodules in lung adenocarcinoma

**DOI:** 10.3389/fcell.2023.1198338

**Published:** 2023-09-07

**Authors:** Xiaofeng Huang, Zhimeng Lu, Xuewei Jiang, Zhe Zhang, Kun Yan, Guiping Yu

**Affiliations:** Department of Cardiothoracic Surgery, Jiangyin Clinical College of Xuzhou Medical University, Jiangyin, China

**Keywords:** single-cell RNA sequencing, lung adenocarcinoma, ground glass nodule, solid nodule, tumor microenvironment, macrophage

## Abstract

**Introduction:** Lung adenocarcinoma (LUAD) is the most prevalent lung cancer. LUAD presents as ground glass nodules (GGN) and solid nodules (SN) in imaging studies. GGN is an early type of LUAD with good prognosis. However, SN exhibits a more malignant behavior than GGN, including worse pathological staging and tumor prognosis. The mechanism leading to the different malignancy levels of GGN and SN remains elusive.

**Methods:** Three patients with GGN and three patients with SN diagnosed with early LUAD were enrolled. The tumor samples were digested to a single-cell suspension and analyzed using 10× Genomic Single-cell ribonucleic acid sequences (scRNA-seq) techniques.

**Results:** A total of 15,902 cells were obtained and classified into nine major types. The tumor microenvironment (TME) was subsequently described in detail. ScRNA-seq revealed that ribosome-related pathways and cell adhesion played similar but distinct roles in the two groups. SN also had more active cell proliferation, enriched cell cycle regulatory pathways, and severe inflammatory responses.

**Conclusion:** We observed changes in the cellular composition and transcriptomic profile of GGN and SN. The study improved the understanding of the underlying mechanisms of lung carcinogenesis and contributed to lung cancer prevention and treatment.

## Introduction

Chest computed tomography (CT) has dramatically increased the detection of early-stage lung adenocarcinoma (LUAD), which have a radiographic appearance of ground glass nodules (GGN) (Travis et al., 2011). Compared to solid nodules (SN), GGNs grow more slowly and have a better prognosis (Zhang et al., 2020). Solid components in pulmonary nodules are closely associated with patient prognosis, with a higher proportion of solids indicating a worse pathological staging and tumor prognosis (Travis et al., 2016). The tumor microenvironment (TME) plays a crucial role in shaping tumor biological and clinical behaviors in addition to cancer cells (Quail and Joyce, 2013). However, whether the TME in GGN is similar to the TME tissue components or invasive components in SN remains unknown. Moreover, the transition of the TME in GGN to the TME in SN has yet to be established. The developing single-cell ribonucleic acid (RNA) sequencing (scRNA-seq) technology can now provide an unbiased transcriptome analysis of single cells and their genetic heterogeneity (Papalexi and Satija, 2018). ScRNA-seq was performed on samples from three patients with GGN and three with SN diagnosed as LUAD. We characterized the GGN and SN ecosystems, identifying differences in their cell types and expression of molecular signatures, providing insightful biological information for further research.

## Materials and methods

### Patients and tissue samples

Tissue samples were collected from six patients who underwent lung surgery at the Department of Thoracic Surgery, Jiangyin Clinical College of Xuzhou Medical University, from January 2020 to December 2020. This study included the following inclusion criteria (Travis et al., 2011): the imaging presentation of GGN in three patients and of SN in the other three patients (Zhang et al., 2020), no lesions other than pulmonary nodule (Travis et al., 2016), no preoperative antitumor treatment (Quail and Joyce, 2013), pathological examination suggestive of LUAD. The detailed clinical information is presented in Supplementary Table S1. This study was approved by the Ethics Committee of Jiangyin Clinical College of Xuzhou Medical University [approval No.2019ER (027)]. All participants provided written informed consent.

### Preparation of single-cell suspensions

Fresh tissues were stored on ice in the sCelLive™ Tissue Preservation Solution (Singleron) within 30 min after surgery. Hanks Balanced Salt Solution (HBSS) was used three times to wash the specimens, mince them into small pieces, and digest them in 3 mL sCelLive™ Tissue Dissociation Solution (Singleron) by Singleron PythoN™ Tissue Dissociation System at 37°C for 15 min. A 40-micron sterile strainer was used to collect and filter the cell suspension. After adding the GEXSCOPE^®^ red blood cell lysis buffer (RCLB, Singleron), the mixture [Cell: RCLB = 1:2 (volume ratio)] was incubated for 5–8 min at room temperature to remove red blood cells. After centrifuging at 300 × g 4°C for 5 min, the mixture was suspended in PBS softly after centrifugation. Finally, Trypan Blue staining was applied to the samples, and cell viability was assessed microscopically.

### RT & amplification & library construction

Single-cell suspensions (2 × 10^5^ cells/mL) with PBS (HyClone) were placed onto a microwell chip using the Singleron Matrix^®^ Single Cell Processing System. Reverse transcription of the mRNA captured by the Barcoding Beads is followed by PCR amplification of the cDNA generated from the Barcoding Bead collection. Sequencing adapters are then ligated to the fragmented cDNA. GEXSCOPE^®^ Single Cell RNA Library Kits (Singleron) were used to construct the scRNA-seq libraries (Dura et al., 2019). Illumina novaseq 6,000 was used to sequence 150 bp paired end reads from individual libraries diluted to 4 nM, pooled, and processed.

### Primary analysis of raw read data

Gene expression matrices were generated using CeleScope (https://github.com/singleron-RD/CeleScope) v1.9.0 pipeline from raw scRNA-seq reads. Raw reads were first processed with CeleScope to remove low-quality reads followed by Cutadapt v1.17 (Martin, 2011) to trim poly-A tails and adapters. Next, cell barcodes and UMI were extracted. Next, we mapped reads to the reference genome GRCh38 (Ensembl version 92 annotation) using STAR v2.6.1a (Dobin et al., 2013). Finally, each cell’s UMIs and gene counts have been acquired with featureCounts v2.0.1 (Liao et al., 2014) software and used to generate expression matrix files for subsequent analysis.

### Quality control, dimension-reduction, and clustering

Python 3.7 was used to perform quality control, dimension reduction, and clustering using scanpyv1.8.2 (Wolf et al., 2018). The expression matrix for each sample dataset was filtered by the following criteria: 1) cells with a gene count less than 200 or with a top 2% gene count were excluded; 2) cells with a top 2% UMI count were excluded; 3) cells with mitochondrial content >50% were excluded; 4) genes expressed in less than 5 cells were excluded. There were 15,902 cells retained after filtering for downstream analyses, with each cell having on average 1,217 genes and 3,433 UMIs. A normalized data matrix was generated by normalizing the raw count matrix by the total number of counts per cell. The top 2000 variable genes were selected by setting flavor = ‘Seurat’. Principle Component Analysis (PCA) was performed on the scaled variable gene matrix, and the top 20 principle components were used to reduce the dimensions and cluster the genes. Cells were split into 21 clusters using the Louvain algorithm with a resolution parameter of 1.2. Cell clusters were visualized by using Uniform Manifold Approximation and Projection (UMAP) {t-Distributed Stochastic Neighbor Embedding (t-SNE)}.

## Statistics and repeatability

Two-tailed Wilcoxon rank-sum tests were used to compare cell distributions between the two groups. Student’s t-test was used to compare gene expression or gene signatures between two groups of cells. Two-tailed Wilcoxon rank-sum tests were performed to compare cell distributions between paired groups 1 and 2. Statistical analyses and presentations were conducted using R. Statistical tests used in figures were shown in figure legends, and statistical significance was set at *p* < 0.05. The exact value of n was shown in the figures, and figure legends and what n represents was shown in the figure legends.

### Differentially expressed genes (DEGs) analysis

To identify differentially expressed genes (DEGs), we used the Seurat FindMarkers function based on the Wilcox likelihood-ratio test with default parameters. We selected the genes expressed in more than 10% of the cells in a cluster, with an average log (Fold Change) value greater than 0.25 as DEGs. The cell types of each cluster were annotated using the DEGs and knowledge from the literature as canonical markers. The expression of markers in each cell type was displayed using heatmaps/dot plots/violin plots generated with the Seurat DoHeatmap/DotPlot/Vlnplot functions. Doublet cells were identified as expressing markers for different cell types and removed manually.

### Cell type annotation

SynEcoSys database was used to identify the cell type identity of each cluster based on canonical markers found in DEGs. In addition, Seurat 3.1.2 was used to generate heatmaps/dot plots/violin plots that illustrated the expression of markers used to identify each cell type.

### Pathway enrichment analysis

The “clusterProfiler” R package 3.16.1 was used to analyze Gene Ontology (GO) and Kyoto Encyclopedia of Genes and Genomes (KEGG) data to investigate DEG functions (Yu et al., 2012). Pathways with p_adj value less than 0.05 were considered significantly enriched. Gene Ontology gene sets, including molecular function (MF), biological process (BP), and cellular component (CC) categories, were used as references. GSVA pathway enrichment analysis used the average gene expression of each cell type as input data (Hänzelmann et al., 2013).

### UCell gene set scoring

Gene set scoring was performed using the R package UCell v 1.1.0 (Andreatta and Carmona, 2021). Based on the Mann-Whitney U statistic, UCell scores rank query genes according to their expression levels in individual cells. The rank-based scoring method of UCell is suitable for large datasets that contain multiple samples and batches.

### scRNA-seq-based CNA detection

The InferCNV package detected the CNAs in malignant cells (cutoff = 0.1, denoise = TRUE, HMM = F, and k_obs_groups = 8) (Kumar et al., 2020). The CNAs of malignant cells were estimated using T cells as baselines. The genes expressed in over 20 cells were sorted according to their loci on each chromosome. Relative expression values were centered at 1 using a 1.5 standard deviation from residual-normalized expression values. The relative expression of each chromosome was smoothened using a sliding window of 101 genes to remove the effects of gene-specific expressions.

### Trajectory analysis

Cell differentiation trajectory was reconstructed using Monocle2 (Qiu et al., 2017) Highly-variable genes (HVGs) was used to sort cells based on their spatial‐temporal differentiation. Next, we used DDRTree to perform FindVairableFeatures and dimension-reduction. Finally, the trajectory was visualized using the plot_cell_trajectory function. Next, CytoTRACE (Gulati et al., 2020) (a computational method that predicts the differentiation state of cells from single-cell RNA-sequencing data using gene Counts and Expression) was used to predict the differentiation potential of monocyte clusters.

### Cell-cell interaction analysis (CellPhoneDB)

CellPhoneDB v2.1.0 (Efremova et al., 2020) analyzed cell-cell interactions based on receptor–ligand interactions between 2 cell types/subtypes. First, the average level of ligand-receptor expression in interacting clusters was calculated by permuting all cell labels 1,000 times randomly. Then, the individual ligand or receptor expression was thresholded based on the average log gene expression distribution across all cell types. Finally, the significant cell-cell interactions were defined as *p*-value <0.05 and average log expression >0.1, visualized with the circle v0.4.10 R package.

### Transcription factor regulatory network analysis

The transcription factor network was constructed by pyscenic v0.11.0 (Van de Sande et al., 2020) using the scRNA expression matrix and transcription factors in AnimalTFDB. The GRNBoost2 model predicts regulatory networks by co-expressing regulators and targets. CisTarget was applied to exclude indirect targets and to search transcription factor binding motifs. Following that, AUCell was used to quantify regulon activity for each cell. Finally, Pheatmap in R was used to visualize top TF regulons with high RSS (Regulon Specificity Score).

## Results

### Single-cell transcriptome analysis of multicellular GGN and SN ecosystems

**FIGURE 1:**
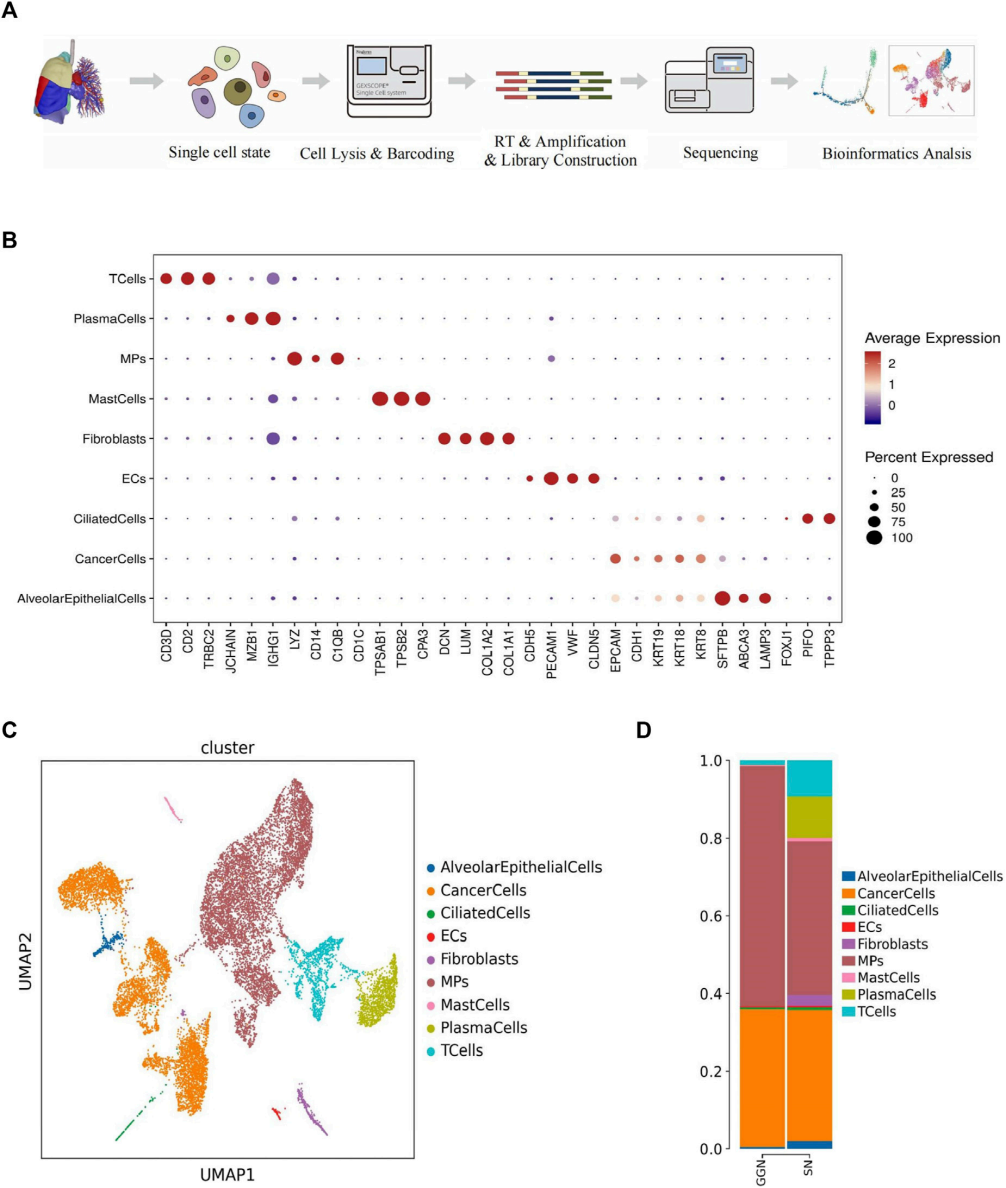
Overview of 15,902 single cells from 3 GGN and 3 SN samples. **(A)**: Workflow for the design of the scRNA-seq experiment and initial data exploration. **(B)**: Bubble plot showing the expression of marker genes for nine major cell types. The dot size is proportional to the fraction of cells expressing the specific genes. The color intensity corresponds to the relative expression of each specific gene. **(C)**: Cell map of the samples shown by UMAP; each dot corresponds to a single cell, and the clusters are labeled by name and color. **(D)**: Bar plot of the relative percentage of cell types in GGN and SN.

A microwell-based scRNA sequence analysis was conducted. We collected 15,902 single isolated cells (4,630 from GGN and 10,392 from SN) from six LUAD samples (Supplementary Figure S1A; Supplementary Figure S6; Supplementary Table S1). The cells were classified into nine major cell types using the Uniform Manifold Approximation and Projection (UMAP) clustering analysis (Figure 1A). SynEcoSys database identified nine cell types annotated with cell type annotation markers, including alveolar epithelial cells (GGN, 22; SN, 227), cancer cells (GGN, 1,644; SN, 3,806), ciliated cells (GGN, 23; SN, 74), endothelial cells (GGN, 10; SN, 43), fibroblasts (GGN, 3; SN, 317), the mononuclear phagocytic system (MPs) (GGN, 2858; SN, 4,468), mast cells (GGN, 14; SN, 90), plasma cells (GGN, 4; SN, 1,205), and T cells (GGN, 52; SN, 1,042) (Figures 1B,C).

The GGN mainly comprised MPs (62%) and cancer cells (36%). However, SN comprised plasma cells (11%) and T cells (9%) in addition to MPs (40%) and cancer cells (34%). Malignant cells were present in both groups in a comparable proportion. The epithelial cells in GGN mainly consisted of cancer cells, whereas SN had a few alveolar epithelial cells (2%) and fibroblasts (3%) (Figure 1D). GGN and SN have complex cellular ecosystems, and their cell proportions differ. Herein, we focused on the functions of important cellular clusters.

### Cancer cells from GGN and SN show different transcriptional characteristics

**FIGURE 2:**
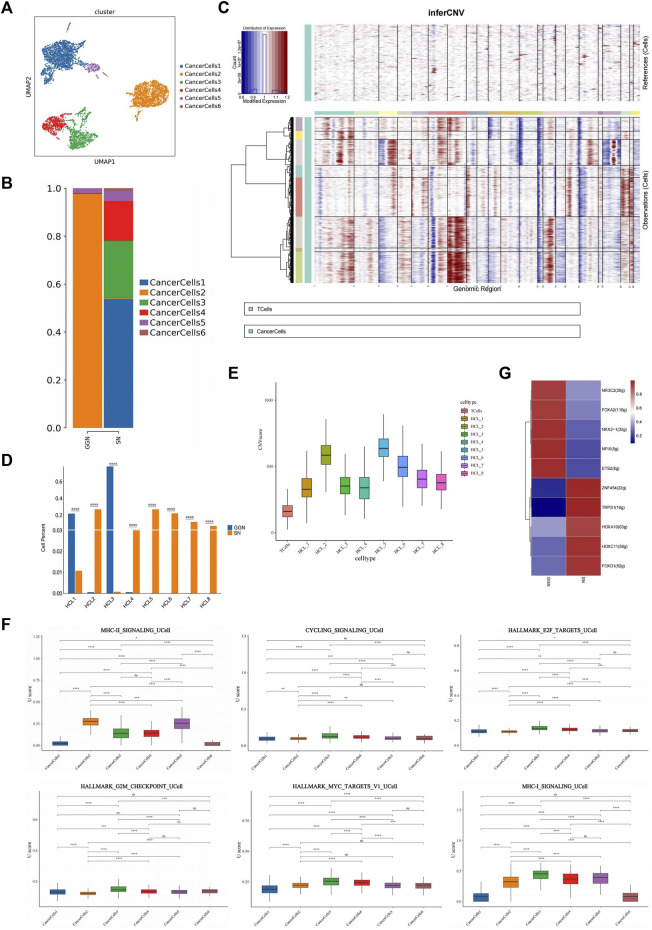
Identification and characterization of cancer cells in GGN and SN. **(A)**: UMAP plot showing cancer cells clustered into six subclusters **(B)**: Bar plot of the relative percentage of cancer cell subclusters in GGN and SN. **(C)**: Heat map showing CNVs Were inferred based on single-cell RNA sequencing data of individual cells from samples. Non-cancer cells were treated as references (top), and CNVs were observed in cancer cells (bottom). The color shows the log2 CNV ratio (HCL_1–8). Red: amplifications; blue: deletions. **(D)**: Bar plot of the relative percentage of cancer cell subclusters in GGN and SN. **(E)**: CNV value of cancer cell subclusters. **(F)**: Box plots of UCell score for different signaling pathways in cancer cell subclusters. Two-sided unpaired Wilcoxon rank-sum test was used for analysis; all differences with *p* < 0.05 are indicated, *p < 0.05, **p < 0.01, ***p < 0.001, ns non-significance. **(G)**:SCENIC analysis of cancer cells (GGN vs SN). Shown are the top 5 upregulated/downregulated transcription factors, respectively.

We detected cancer cells using markers typical of LUAD and alveolar epithelium (EPCAM, CDH1, KRT19, KRT18, KRT8) (Figure 1B). A total of 5,450 cancer cells were categorized into six subclusters (GGN, 1,644; SN, 3,806) (Figure 2A). The clustering revealed group specificity, with CancerCells (CaC) 2 predominantly present in GGN, whereas SN primarily comprised CaC1, CaC3, and CaC4. A single subcluster dominated the presence of cancer cells in GGN. However, the cancer cells in SN revealed a mixture of multiple subclusters with no obvious dominant cluster. Patients with SN had greater inter-tumour heterogeneity than those with GGN (Figure 2B).

Cancer cells exhibited a higher copy number variation (CNV) than immune cells. CNV is a general term used to describe a molecular phenomenon of genome sequence duplication, The analysis of CNVs is an important aspect of tumor molecular diagnosis (Pös et al., 2021). Extensive chromosome 13 deletion was observed in cancer cell samples (Figure 2C). Human chromosome 13 (HSA13), which comprises 1,381 genes, 41 novel genes, and 477 pseudogenes, has the lowest proportion of repetitive sequences and is connected with the initiation and progression of various human cancers and disorders (Bailey et al., 2002). The prognosis of patients with LUAD is adversely affected by copy number deletion of chromosome 13 (Han et al., 2019). The CNV values divided cancer cells into eight subclusters (HCL_1–8) (Supplementary Figure S1B). GGN mainly comprised HCL_1 and HCL_3, whereas SN primarily comprised HCL_2, HCL_4, HCL_5, HCL_6, HCL_7, and HCL_8 (Figure 2D). Chromosomes 16, 18, and 20 in the GGN cancer cell subclusters underwent significant amplification. In addition, chromosomes 1 and 7 of the SN cancer cell subclusters underwent significant amplification, whereas chromosomes 10 and 15 underwent deletion (Figure 2C). The CNV values of HCL_2, HCL_5, and HCL_6 were significantly higher than those of other subclusters (Figure 2E), indicating that SN cancer cells have a higher degree of CNV than GGN cancer cells. Studies have proved that CNV variation is related to tumor malignancy and prognosis of patient (Pös et al., 2021), suggesting that SN tumor cells are more malignant.

GGN cancer cells express significantly higher levels of surfactant-related proteins than SN cancer cells, such as surfactant protein (SFTP) A1, SFTPA2, and SFTPB. In addition, cancer cells in SN upregulated MT-RNR2, DST, and MALAT1 (Supplementary Figure S1C). Furthermore, cancer cells in GGN highly expressed alveolar type II (AT2) cell-related marker genes (SFTPA1, SFTPA2, SFTPB, ABCaC3), suggesting that cancer cells in GGN might originate from AT2 cells. However, the alveolar type I (AT1) cell marker genes PDPN or AGER was not highly expressed in either group (Supplementary Figure S1D) (Jacob et al., 2017; Evans and Lee, 2020), suggesting that AT1-like cells had a low expression level in both samples.

Gene Ontology (GO) pathway enrichment analysis revealed that cancer cells in GGN upregulate ribosome-related pathways, establish protein localization to the endoplasmic reticulum, cotranslational protein targeting to membrane, Major Histocompatibility Complex (MHC) class II protein complex binding, and other pathways. In addition, the Kyoto Encyclopaedia of Genes and Genomes (KEGG) analysis revealed ribosome-related pathway enrichment ((Supplementary Figure S2A). Ribosomal protein (RP) family-related genes (RPS21,RPS2,RPS18,RPLP1,RPL5,RPL13 A) were significantly upregulated in GGN (Supplementary Figure S1E). Thus, ribosome-related pathways play a crucial role in GGN tumourigenesis. However, RNA splicing, cell cycle phase transition regulation, transcription coregulator activity, and other pathways were upregulated in cancer cells in SN. The KEGG enrichment analysis also revealed enrichment of the pathways associated with spliceosome and protein processing in the endoplasmic reticulum (Supplementary Figure S2B). RNA splicing-related pathways were enriched in SN. Aberrant RNA splicing factor expression in oncogenes and cancer suppressor genes can regulate post-transcriptional mechanisms to promote tumor growth (David et al., 2010; Hsu et al., 2015). According to the gene set variation analysis (GSVA) and UCell gene set score analysis, CaC2 scored higher in MHC-II_SIGNALLING; CaC1, CaC3, and CaC4 scored higher in cell cycle regulation pathways such as CYCLING_SIGNALLING, HALLMARK_E2F_TARGETS, and HALLMARK_G2M_CHECKPOINT compared with CaC2; CaC3 and CaC4 scored higher in the HALLMARK_MYC_TARGETS_V1 and MHC-I_SIGNALLING (Figure 2F; Supplementary Figure S2C). Therefore, compared with SN, GGN was enriched in the MHC-II_SIGNALLING pathway. SN was enriched in cell cycle regulation pathways and the MHC-I_SIGNALLING pathway. The above results suggest different functional patterns of the two groups of cancer cells, resulting in differences in proliferation and aggressiveness.

Single-cell regulatory network inference and clustering analysis (SCENIC) showed significant upregulation of E26 transformation-specific transcription factor 2 (ETS2) in GGN and transcriptional repressor erythroid transcription factor binding 1 (TRPS1) in SN (Figure 2G). ETS2, an ETS transcription factor family oncogene, inhibits lung cancer cells’ growth, migration, and invasion by suppressing mesenchymal-epithelial transition (MET) expression (Kabbout et al., 2013). TRPS1, a GATA family transcriptional regulatory factor, has been shown to induce tumor angiogenesis, affect VEGFA expression, and promote tumor cell proliferation in tumors (Hu et al., 2014). TRPS1 can also induce MET to increase malignant cell migration and invasion through ZEB2 regulation (Stinson et al., 2011).

An analysis of the trajectory of six cancer cells was performed to determine the developmental relationship between cancer cells in GGN and SN. The dominant subclusters of cancer cells in GGN were concentrated at the tail end of the trajectory. In contrast, cancer cells in SN were scattered in the anterior-middle part of the trajectory. Therefore, cancer cells in SN were more dispersed and differentiated earlier in the trajectory than in GGN, and the cancer cell stemness in the former might be stronger (Supplementary Figure S2D). In addition, expressions of C16orf89, CD74, CD55, and CCND1 were significantly upregulated at the end of the trajectory as the trajectory developed (Supplementary Figure S2E). CD74 is a type II transmembrane protein with various biological functions that promote the angiogenesis of lung cancer cells when co-expressed with macrophage migration inhibitory factor (MIF) (McClelland et al., 2009). In addition, CD74 can form oncogenic fusion genes with NRG1 to promote the progression of lung cancer cell (Murayama et al., 2016). CD55 and CCND1 are reportedly associated with cancer progression, as demonstrated in liver cancer (Meng et al., 2017; Nie et al., 2020).

### Varying distribution of MPs subtypes between GGN and SN

**FIGURE 3:**
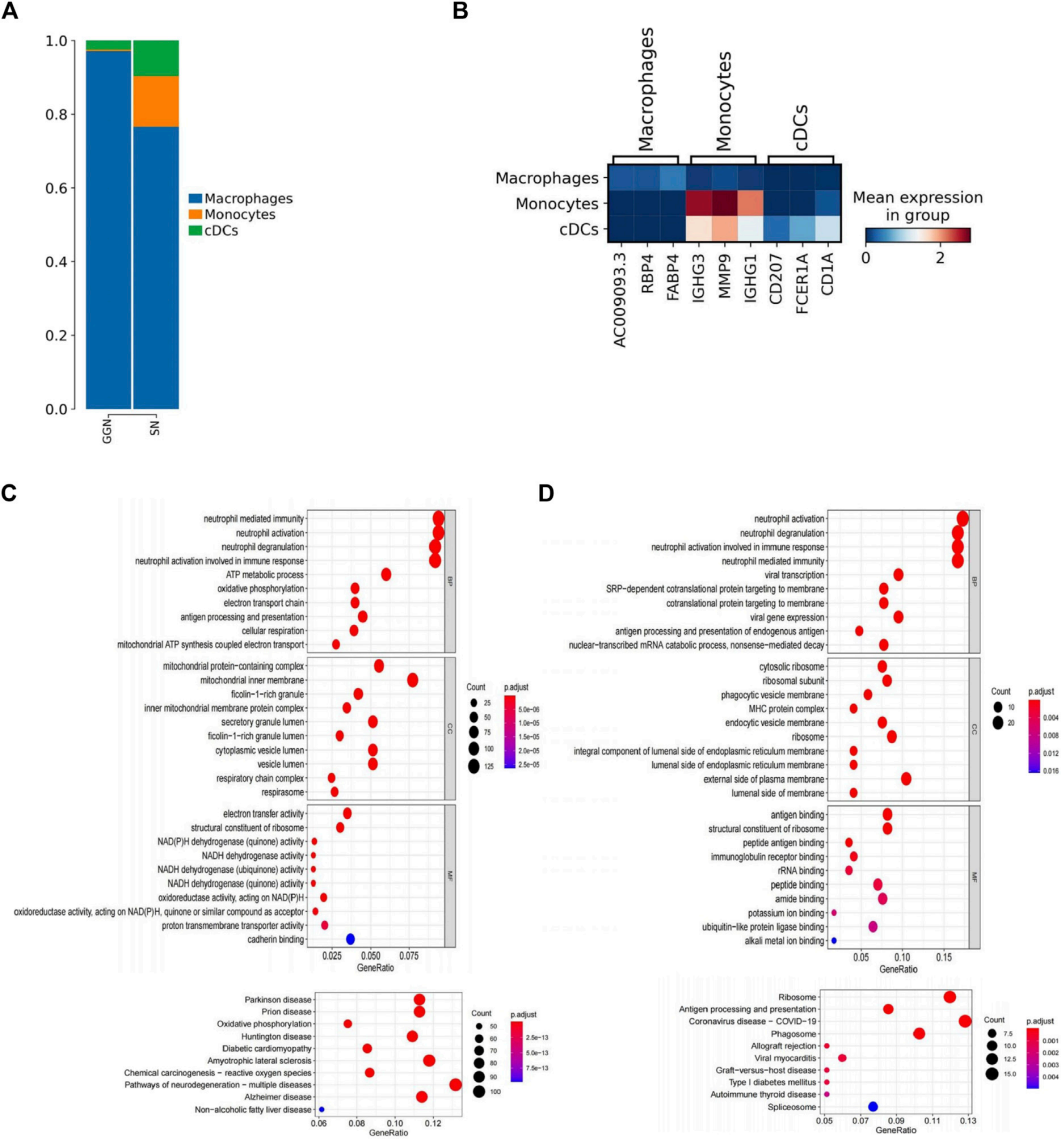
Detailed description of MPS **(A)**: Bar plot of the relative percentage of MPS subclusters in GGN and SN. **(B)**: Heat map showing marker genes of three MPS subclusters. **(C, D)** The bubble plots show significantly enriched GO and KEGG pathways of cDCs (C) and monocytes (D) in SN. The color of the bubbles represents the values of significance, and the size represents the number of genes enriched in the pathway.

MPs were identified by marker genes (LYZ, CD14, C1QB, CD1C) (Figure 1B). A total of 6,726 MPs (2,858 in GGN and 4,468 in SN) were divided into three subclusters: macrophages, monocytes, and conventional dendritic cells (cDCs). Among them, macrophages accounted for most of the subclusters. In GGN, macrophages were almost dominant, and a small number of cDCs(3%) were also included. However, in addition to most macrophages, SN comprised a certain percentage of monocytes (14%) and cDCs (10%) (Figure 3A). CDCs (CD207+, FCER1A+, CD1A+) (Figure 3B) were more enriched in SN. The GO and KEGG enrichment pathway analyses revealed that the upregulated enrichment pathways of cDCs in SN included neutrophil-related immune response and oxidative phosphorylation pathways (Figure 3C). In SN, monocytes (IGHG3+, MMP9+, IGHG1) (Figure 3B) accounted for approximately 14% of the MPs. In contrast with GGN, monocytes in SN are recruited extensively into the tumor tissue. They are differentiated into macrophages and dendritic cells, altering the TME and promoting immune evasion, angiogenesis, and metastatic growth (Olingy et al., 2019). GO and KEGG pathway analysis findings indicate that monocytes in the SN are up-regulating neutrophil-related immune response, ribosome, and antigen processing and presentation pathways (Figure 3D). SN comprises abundant monocytes and cDCs and demonstrates, Moreover, both cells were enriched in neutrophil-related immune response pathways. SN showed stronger immune activity.

### Macrophages in SN exhibit stronger proliferative capacity and inflammatory response

**FIGURE 4:**
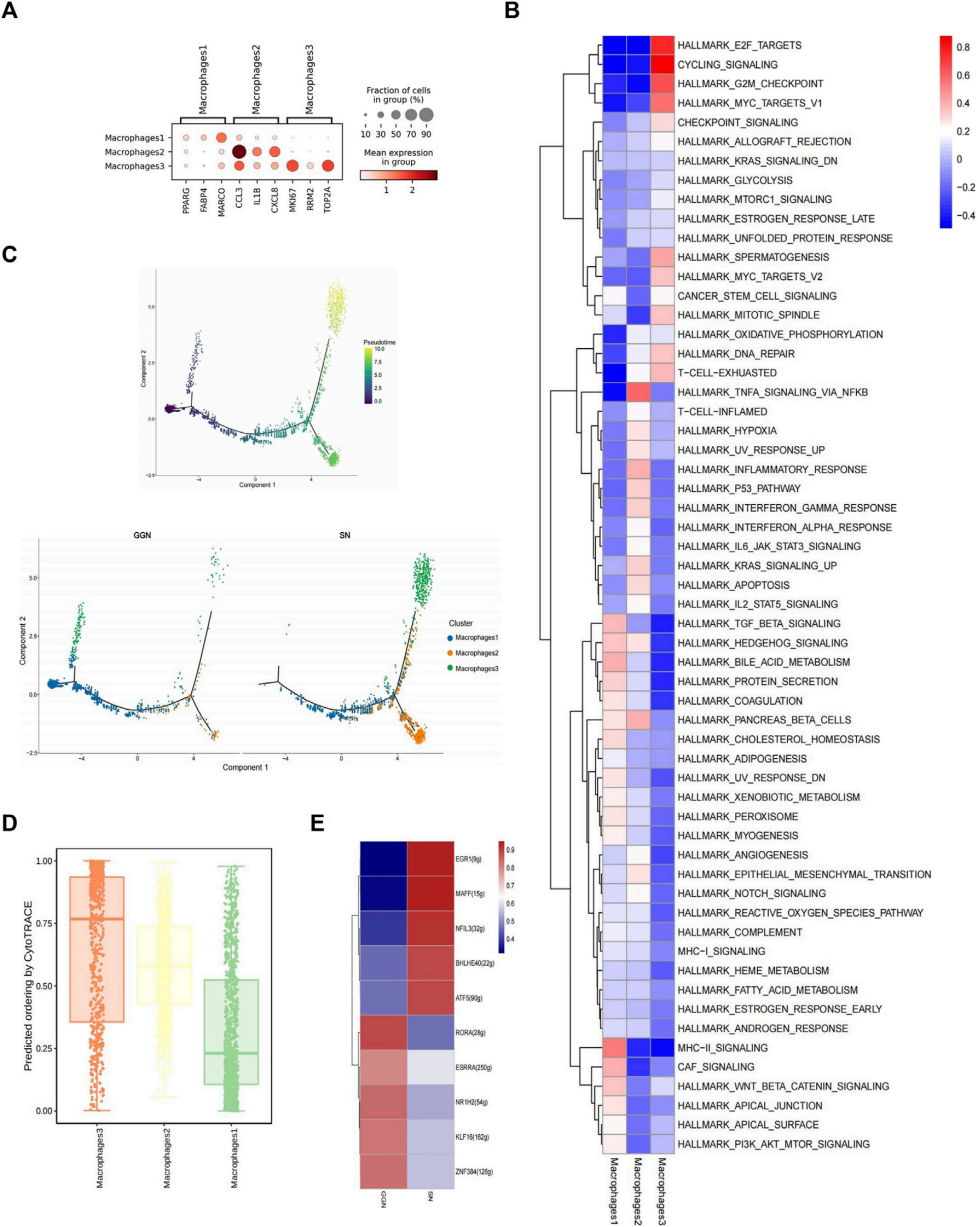
Different composition and characteristics of macrophages between GGN and SN. **(A)**: Bubble plot showing marker genes of macrophage subcluster. The color of the bubbles represents the average expression level of the gene, and the size represents a fraction of cells. **(B)**: Heat map showing the difference in metabolic pathways scored by GSVA of macrophage subclusters. **(C)**: Differentiation trajectory of cancer cells was predicted by monocle 2. **(D)**: Box plot showing cytotrace scores of macrophage subclusters, ranked from top to bottom by the median value. **(E)**: SCENIC analysis of macrophages (GGN vs SN). Shown are the top 5 upregulated/downregulated transcription factors, respectively.

Macrophages, one of the important components of the innate immune system, are crucial for normal homeostasis and disease development and are closely associated with tumor development (Murray and Wynn, 2011). MPs comprise abundant macrophages (Figure 3A). We identified three macrophage subclusters, namely, alveolar-resident macrophage 1 (Mac1) (PPARG+, FABP4+, MARCO+), inflammatory cytokine-enriched macrophage 2 (Mac2) (CCL3+, IL1b+, CXCL8+), and proliferative macrophage 3 (Mac3) (MKI67+, RRM2+, TOP2A+) (Ma et al., 2022) (Figure 4A). GGN mainly comprised Mac1 (89%), as well as smaller quantities of Mac2 (5%) and Mac3 (6%). However, SN primarily comprised Mac1 (41%) and Mac2 (46%) and a smaller quantity of Mac3 (13%) (Supplementary Figure S3A).

According to the combined analysis of GSVA analysis and UCell gene set score, Mac1 scored higher in the HALLMARK_BILE_ACID_METABOLISM pathway; Mac3 scored higher in the cell cycle regulatory pathways such as HALLMARK_G2M_CHECKPOINT, CYCLING_SIGNALLING, HALLMARK_E2F_TARGETS, and HALLMARK_MYC_TARGETS_V1 (Figure 4B; Supplementary Figure S3A); Mac2 scored high in the HALLMARK_TNFA_SIGNALLING_VIA_NFKB (Figure 4B; Supplementary Figure S3B). In addition, Mac2 highly expressed inflammatory factors such as IL1B, CCL3, CCL4, CXCL2, and CXCL8 (Supplementary Figure S3C), suggesting a more severe inflammatory response in SN, which may act through the TNFA_SIGNALLING_VIA_NFKB pathways. However, Mac3 highly expressed proliferation-related genes, such as MKI67, RRM2, and TOP2A (Supplementary Figure S3C). It might promote tumor-associated macrophage proliferation and tumor growth by regulating the cell cycle through the above pathways (Murray and Wynn, 2011).

According to the GO and KEGG enrichment analyses in both groups, pathways such as neutrophil-related immune response, ribosome, RNA splicing, RNA catabolic process, and mRNA catabolic process were upregulated in SN (Supplementary Figure S4A), pathways such as upregulation of neutrophil-related immune response, focal adhesion, cell-substrate junction were upregulated in GGN. No significant KEGG pathway enrichment was observed in GGN (Supplementary Figure S4B). Increased pro-inflammatory cytokines and chemokines, such as IL1B, CCL3, CCL4, CXCL2, and CXCL8, were secreted by macrophages in SN (Supplementary Figure S4C). These cytokines can promote the occurrence and development of inflammatory response, so it could be inferred that TME in SN has a more severe inflammatory response.

The Monocle2 package performed unsupervised trajectory analysis of macrophage transcriptional changes. It showed that Mac1 might differentiate into Mac2 and Mac3. Since Mac1 were mainly expressed in GGN while Mac2,3 were mainly expressed in SN, More Mac1 might be induced conversing to Mac2 and Mac3 in SN (Figure 4C). The greatest differentiation potential of proliferating Mac3 was suggested by CytoTrace analysis (Figure 4D). In addition, the tumor-associated gene Activating Transcription Factor 3 (ATF3) was upregulated, and the oncogene Aldehyde Dehydrogenase 2 (ALDH2) was downregulated as the trajectory progressed (Supplementary Figure S4D).

SCENIC analysis revealed significant Early growth response factor 1 (EGR1) upregulation in SN and Nuclear receptor subfamily 1 Group H Member 2(NR1H2) in GGN (Figure 4E). EGR1 regulates cell proliferation, apoptosis, immune cell activation, and stromal degradation and is closely associated with tumorigenesis (Li et al., 2019). NR1H2 suppresses inflammatory genes in macrophages (A-González NCastrillo, 2011), activates liver X receptors, and inhibits cancer cell growth, including colorectal cancer (Liang et al., 2019).

In summary, macrophages in SN exhibited greater proliferative capacity and inflammatory response than those in GGN.

### Significant immune cell enrichment in SN

**FIGURE 5:**
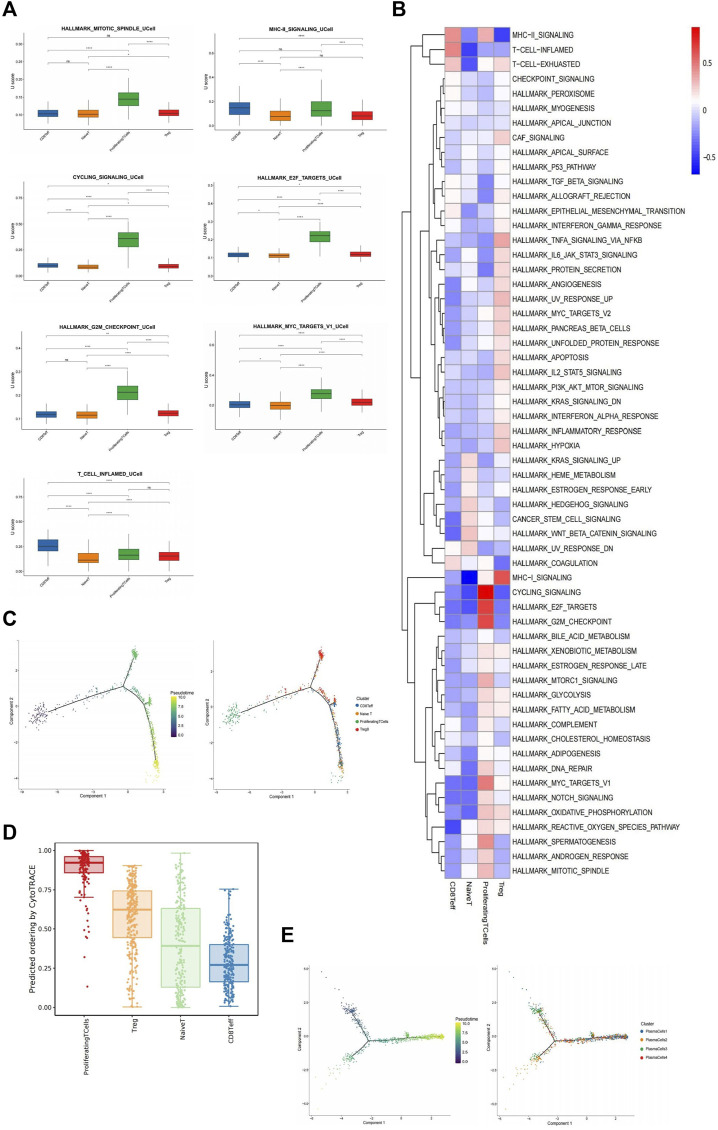
Immune cells play a greater role in SN. **(A)**: Box plots of UCell score for different signaling pathways in T cell subclusters. Two-sided unpaired Wilcoxon rank-sum test was used for analysis; all differences with *p* < 0.05 are indicated, *p < 0.05, **p < 0.01, ***p < 0.001, ns non-significance. **(B)**: Heat map showing the difference in metabolic pathways scored by GSVA of T cell subclusters. **(C)**: Differentiation trajectory of T cells was predicted by monocle 2. **(D)**: Box plot showing cytotrace scores of macrophage subclusters, ranked from top to bottom by the median value. **(E)**: Differentiation trajectory of four plasma cell subclusters was predicted by monocle 2.

1,094 T cells were identified based on labeled marker gene expression (CD3D, CD2, TRBC2) (Figure 1B). SN exhibited increased T cell enrichment, with decreased enrichment exhibited by GGN (Figure 1D). T cells were subdivided into four clusters, which were as follows: CD8 +T effector cell (CD8Teff), native T cell (NaiveT), proliferating T cell, and regulatory T cell (Treg). According to the GSVA analysis and UCell gene set score, proliferating T cells scored higher in HALLMARK_MITOTIC_SPINDLE, MHC-II_SIGNALING, and cell cycle regulation pathways, such as CYCLING_SIGNALLING, HALLMARK_E2F_TARGETS, HALLMARK_G2M_CHECKPOINT, HALLMARK_MYC_TARGET_v1 (Figures 5A,B). However, CD8Teff scored higher in the T_CELL_INFLAMED pathway (Figures 5A,B). According to the pseudotime analysis, proliferating T cells appeared first in the early stage of the trajectory, and the remaining three clusters were distributed in the middle and late stages (Figure 5C). In addition, CytoTrace analysis revealed the greatest differentiation potential of proliferating T cells (Figure 5D), suggesting that proliferating T cells promote the development of inflammation and the formation of TME by regulating the cell cycle.

Based on differential gene expression, the 1,209 plasma cells identified by the marker genes (JCHAIN, MZB1, IGHG1) (Figure 1B) were divided into four clusters: PlasmaCells1 (PC1) (SCGB3A1+, ENAM+, JCHAIN+), PlasmaCells2 (PC2) (CD3G+, CD52^+^, CST+), PlasmaCells3 (PC3) (Z93241.1+, NR4A1+, HSP90AA1+), and PlasmaCells4 (PC4) (EGR1+, IER2+, FOS+) (Supplementary Figure S5A). Plasma cells were abundantly enriched mainly in SN but were minimally expressed in GGN (Figure 1D). According to the combined analysis of GSVA and UCell gene set scores, PC2 scored highest in the T_CELL_INFLAMED and HALLMARK_MITOTIC_SPINDLE pathways (Supplementary Figures S5B,C). According to the pseudotime analysis, PC2 and PC4 clusters were distributed across all time segments, PC1 was primarily distributed in the posterior segment, and PC3 was primarily distributed in the anterior segment (Figure 5E), suggesting that the other plasma cell subclusters may be formed by PC1 differentiation, and PC3 may be in the mature terminal differentiation stage.

In summary, SN significantly enriched immune cells and formed a complex immune microenvironment.

### Cancer cell-associated interactions suggest different patterns of tumor development between GGN and SN

**FIGURE 6:**
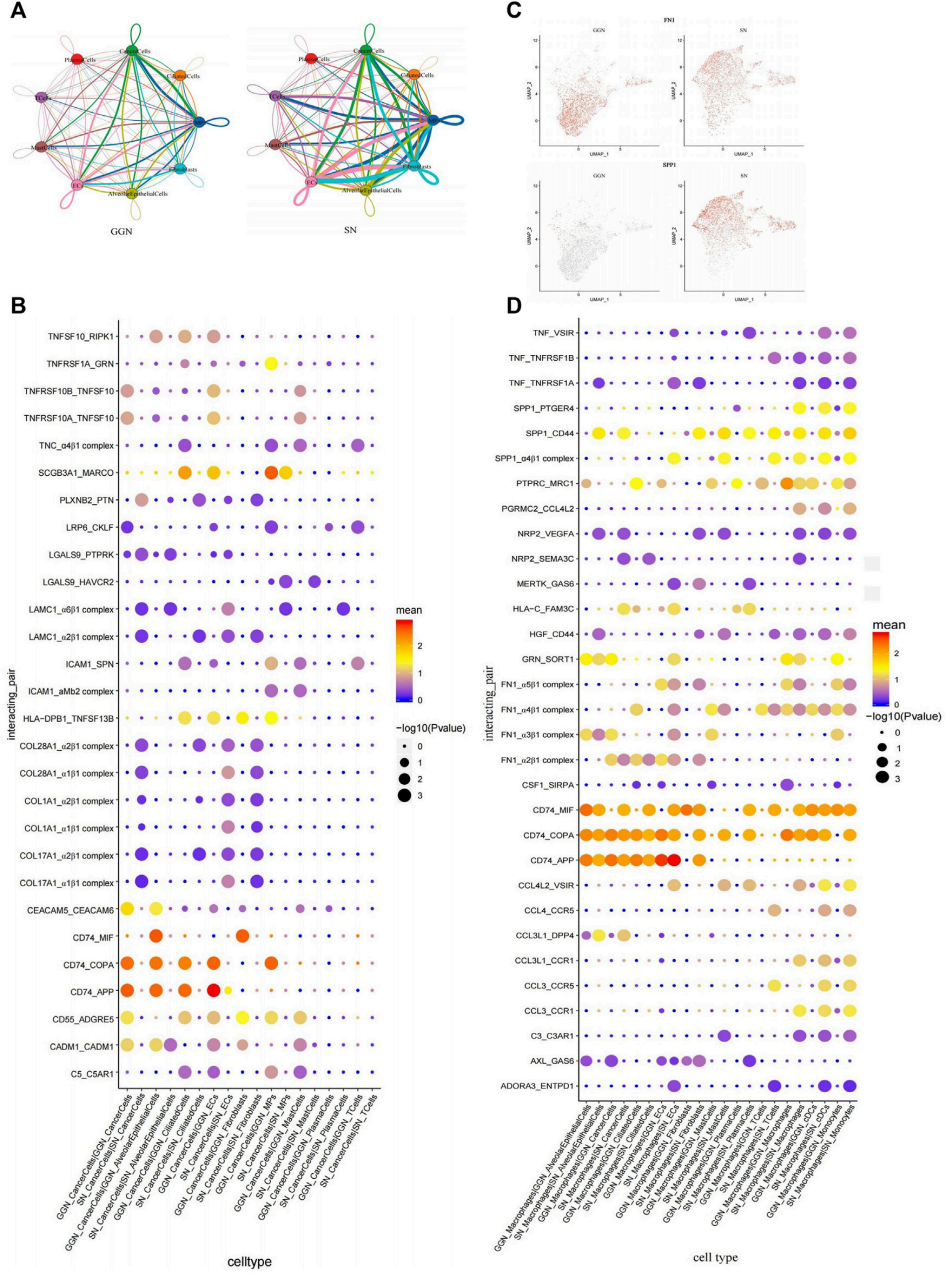
Intercellular interactions between GGN and SN. **(A)**: Interaction network diagram of cell types between GGN (left) and SN (right), the network node represents the cell type, the thickness of the network edge represents the total number of ligand and receptor pairs, and the line color represents the ligand cell type. **(B)**: Bubble plot showing ligand-receptor pairs of cancer cells in GGN and SN. Bubbles are labeled by −log10(P) (size) and average expression level of ligand–receptor pairs (color). *X*-axis: cell types of the two groups, *Y*-axis: ligand-receptor pairs. **(C)**: The expression of FN1 and SPP1 of macrophages in GGN and SN. **(D)**: Bubble plot showing ligand-receptor pairs of macrophages in GGN and SN. Bubbles are labeled by −log10(P) (size) and average expression level of ligand–receptor pairs (color). *X*-axis: cell types of the two groups, *Y*-axis: ligand-receptor pairs.

The significant differences in the gene expression patterns of cancer cells between GGN and SN might result in different interactions between cancer cells and TME. Therefore, CellphoneDB was used to study cancer cell-specific interaction pairs in different fractions. According to the cellular interaction pair network diagram, the number of intercellular pairs was higher in SN than in GGN, indicating that cancer cells in SN interact more with other cells (Figure 6A).

The adhesion-related molecules ADGRE5 and ICaCM1 were significantly upregulated in GGN (Supplementary Figure S5D). They interacted with the corresponding receptor CD55 and SPN (Figure 6B) and promoted tumor invasion and metastasis through the adhesion of cancer cells to normal cells (Ksiazek et al., 2010; Meng et al., 2017). Carcinoembryonic antigen cell adhesion molecule 5 (CEACAM5)-CEACAM6 interaction was significant in GGN (Figure 6B). In addition, CEACAM6 might also be involved in key cellular events such as tumor migration and invasion in GGN through adhesion (Blumenthal et al., 2005; Cameron et al., 2012). TNFRSF10 and its receptor have obvious interaction in GGN (Figure 6B), suggesting inflammation and apoptosis between cancer cells, cancer cells, and endothelial cells, and between plasma cells in GGN (Allen and El-Deiry, 2012; Cucolo et al., 2022). CD74 was significantly upregulated in cancer cells in GGN (Supplementary Figure S5D) and was strongly associated with APP and COPA in alveolar epithelial cells, ciliated cells, and endothelial cells (Figure 6B). CD74_APP and CD74_COPA interactions were less commonly reported. However, their high enrichment suggests their crucial role in GGN development and warrants further research.

SN is enriched in integrin-associated complex interactions, which form complexes with collagen family genes (COL28A1, COL1A1, COL17A1) and human laminin γ1 (LAMC1) (Figure 6B). Integrins, the typical adhesion molecules in cancer cells, bind to collagen to promote the occurrence and development of tumors. Different collagen types can bind to various integrins in multiple signaling pathways in cancer cells (Xu et al., 2019). For example, LKB1 is involved in the HIF/LOX pathway through collagen type IV and β1 integrins resulting in enhanced lung cancer cell proliferation and invasiveness (Gao et al., 2010). Integrin α2β1 mediates the adhesion of lung cancer cells to type IV collagen, enhances cancer cell proliferation, and promotes the metastasis of lung cancer cells to the liver (Burnier et al., 2011). In addition, synergistic effects of integrins with LAMC1 promote cell migration and invasion, regulate cell adhesion and motility to promote tumor progression, and are associated with poor prognosis (Zacapala-Gómez et al., 2020). In a word, cell adhesion plays an important role in SN.

In summary, our data suggested that complex interactions existed between cancer cells and cells of various compositions. And there were significant differences between GGN and SN.

### The interaction characteristics of macrophages between two groups reflect changes in the immune environment

Macrophage subclusters differed in their functional distribution and the cellular interactions between SN and GGN. Fibronectin 1 (FN1), which is highly expressed by GGN macrophages (Figure 6C), could form complexes with integrin subunits of other cells (FN1_α2β1 complex, FN1_α3β1 complex, FN1_α4β1 complex, FN1_α5β1 complex) (Figure 6D), which promoted cancer cell migration through adhesion (Huaman and Ogunwobi, 2020). Secreted phosphoprotein 1 (SPP1), highly expressed in SN, interacted significantly with CD44, PTGER4, and α4β1 (Figures 6C,D). SPP1, a multifunctional secretory acidic glycoprotein highly expressed in lung cancer, mediates macrophage polarisation and tumor immune evasion (Hu et al., 2005; Zhang et al., 2017). A tight association between Neuropilin 2 (NRP2) and VEGFA and SEMA3C was observed in SN. NRP2 regulates lymphatic vessel growth and promotes tumor metastasis to the lymphatic pathways (Raimondi and Ruhrberg, 2013). SN comprises extensive cell adhesion-related interactions like HGF_CD44 (Xu et al., 2021). In contrast with GGN, CCL4 and CCL3 interact significantly with their receptors in SN (CCL4L2_VSIR, CCL4_CCR5, CCL3L1_DPP4, CCL3L1_CCR1, CCL3_CCR5, CCL3_CCR1) (Figure 6D). The CC chemokines, CCL4 and CCL3, act alone or together by regulating the tumor immune microenvironment to promote tumor progression (Mukaida et al., 2020; Ntanasis-Stathopoulos et al., 2020). TNF-related interactions such as TNF_VSIR (Shapouri-Moghaddam et al., 2018) and CD74_MIF interactions are enriched in SN (Su et al., 2017), exhibiting more inflammation-related tumor features. Complex interactions exist between macrophages and cells of various compositions, significantly different between GGN and SN.

## Discussion

GGN is an inert pathological subtype of LUAD with slow progression. GGN is less malignant than SN. Therefore, patients with GGN have a better prognosis than those with SN. Additionally, the clinical treatment strategies differ (Mao et al., 2019; Zhang et al., 2020). The present study comprehensively characterizes cellular profiles and transcriptomics in GGN and SN samples. It is revealed that the distinct cellular features and TMEs of the cell subtypes differ when the dynamic changes in their composition, expression of the molecular signatures, and intercellular interactions are compared. These findings help us understand lung cancer onset and progression mechanisms.

Cancer cells have the greatest heterogeneity compared with other cell types. Nevertheless, the proportion of cancer cells was similar in both groups. However, the higher CNV values of cancer cells in SN compared with cancer cells in GGN indicated a higher variability, which is consistent with the findings reported by Lu et al. (2020). This might be owing to the higher tumor mutational load in SN than in GGN (Chen et al., 2021). In GGN, the cancer cells comprise one major subcluster. However, SN comprises multiple tumor subclusters. In addition, GGN cancer cells expressed AT2-associated genes, suggesting they may be derived from AT2 cells.

Compared with GGN, SN upregulated genes promote cell proliferation and migration like EGR1 and TRPS1, and downregulated oncogenes like NR1H2 and ETS2. In cancer cell analysis, ribosome-related pathways were significantly enriched in GGN. Ribosomes are important organelles for protein synthesis. In addition to performing protein translation, ribosomes co-regulate cancer cell growth and proliferation through the RAS/RAF/MEK/ERK, MYC, and PI3K/AKT/mTOR pathways (Woods et al., 2015). However, in macrophage analysis, ribosome-related pathways were more enriched in SN than in GGN, which might be associated with inflammation in lung cancer. Several studies have reported that tumors, including lung cancer, are strongly associated with inflammation, and upregulation of the ribosome biogenesis rate might be involved in tumor transformation in tissues affected by chronic inflammation, upregulation of rRNA transcription results in increased MDM2-mediated degradation of P53 proteasome, which reduces P53 expression and promotes tumorigenesis (Donati et al., 2011). Cell cycle regulation-related pathways in SN are significantly enriched in cancer cells, macrophages, and T cells. The G2M_checkpoint pathway plays a crucial role in various human tumors (Evan and Vousden, 2001) and regulates cell growth and apoptosis in ovarian cancer (Zaffaroni et al., 2001) and colon cancer (Yang et al., 2018). E2F_targets (De Meyer et al., 2009) and MYC_targets_V1 (Stine et al., 2015) also involve tumor therapy, drug resistance, immune evasion, and progression. Tumor-associated macrophages are involved in the cell cycle regulation of cancer cells through M2 polarisation (Yoshikawa et al., 2012). The above pathways might be involved in SN development. Considering that immune cells, such as T and plasma cells, are mainly enriched in SN, gene expression of cancer cells and macrophages and pathway analysis suggested a more severe inflammatory response in SN than GGN. Therefore, it is speculated that the inflammatory response is a differentiating factor between GGN and SN.

The present study confirmed region-specific cellular interactions. Significant intercellular interactions between the two groups were screened for analysis. Integrins exerted adhesion interactions in both groups. However, certain differences were observed. In GGN, macrophages interacted with other cells via FN1 and integrin subunit pairing. However, in SN, cancer cells interact with other cells through a complex formed by collagen family genes, LAMC1, and integrin subunits. A clear association of CD44, PTGER4, and α4β1 in SN macrophages, plasma cells, cancer cells, and T cells was observed with upregulated SPP1 in macrophages. The activated interaction pairs were associated with macrophage polarisation and tumor immune evasion (Zhang et al., 2017). Inflammation-associated cellular interaction pairs were observed in both groups. However, the interactions were more pronounced in SN. Thus, SN and GGN exhibit similar but distinct specific interactions.

Our study has certain limitations. We could only partially describe all cell types, subtypes, and phenotypes in one report. Therefore, only certain key observations were reported herein, and further studies at a more comprehensive and deeper level need to be conducted.

## Conclusion

In summary, the complex cellular and ecosystem processes in patients with lung cancer were characterized by the scRNA-seq technique, and GGN and SN exhibited similar but different specific TME. Macrophages might also contribute to the differences between SN and GGN in addition to cancer cells. In general, this study contributes to the understanding of LUAD and the discovery of new therapeutic targets.

## Data Availability

The data presented in the study are deposited in the Genome Sequence Archive (Genomics, Proteomics & Bioinformatics 2021) in National Genomics Data Center (Nucleic Acids Res 2022), China National Center for Bioinformation/Beijing Institute of Genomics, Chinese Academy of Sciences, accession number (GSA-Human: HRA005270).
