# Supplementary material for: Single-cell RNA sequencing reveals distinct tumor microenvironment of ground glass nodules and solid nodules in lung adenocarcinoma
Source: Front Cell Dev Biol. 2023 Sep 7;11:1198338. doi: 10.3389/fcell.2023.1198338 (PMC10513029; doi:10.3389/fcell.2023.1198338)
Supplement: Supplementary file 1 [file DataSheet1.docx]

Single-Cell RNA Sequencing Reveals Distinct Tumor Microenvironment of ground glass nodules and solid nodules in lung adenocarcinoma

**Xiaofeng Huang^1^, Zhimeng Lu^1†^, Xuewei Jiang^2^ , Zhe Zhang^2^, Kun Yan^2,3^, Guiping Yu^2,*^**

*** Correspondence:** Guiping Yu [xiaoyuer97103@163.com](mailto:xiaoyuer97103@163.com)

**Supplementary Table S1. Detailed clinical information for 6 patients**

| **Individual**  **ID** | **Gender** | **Age** | **Surgery date** | **Tumor location** | **Surgical type** | **Predominant**  **subtype** | **Tumor**  **diameter (cm)** | **pTNM** | **Stage** | |
| --- | --- | --- | --- | --- | --- | --- | --- | --- | --- | --- |
| GGN1 | male | 67 | 2020/02/24 | LUL | Segmentectomy | Acinar | 2.1 | T1cN0M0 | IA | |
| GGN2 | female | 59 | 2020/12/12 | RLL | Segmentectomy | Acinar | 1.9 | T1bN0M0 | IA | |
| GGN3 | female | 56 | 2020/12/26 | RUL | Segmentectomy | Acinar | 1.7 | T1bN0M0 | IA | |
| SN1 | female | 62 | 2020/03/03 | RLL | Lobectomy | Acinar | 2.5 | T1cN0M0 | IA | |
| SN2 | male | 63 | 2020/03/03 | RUL | Lobectomy | Papillary | 2.1 | T1cN0M0 | IA | |
| SN3 | male | 51 | 2020/03/12 | LLL | Lobectomy | Acinar | 2.2 | T1cN0M0 | IA | |
| Note: LUL,left upper lobe; LLL, left lower lobe; RUL,right upper lobe; RML, right middle lobe; RLL,right lower lobe | | | | | | | | | |  |

**Supplementary Figures**

**
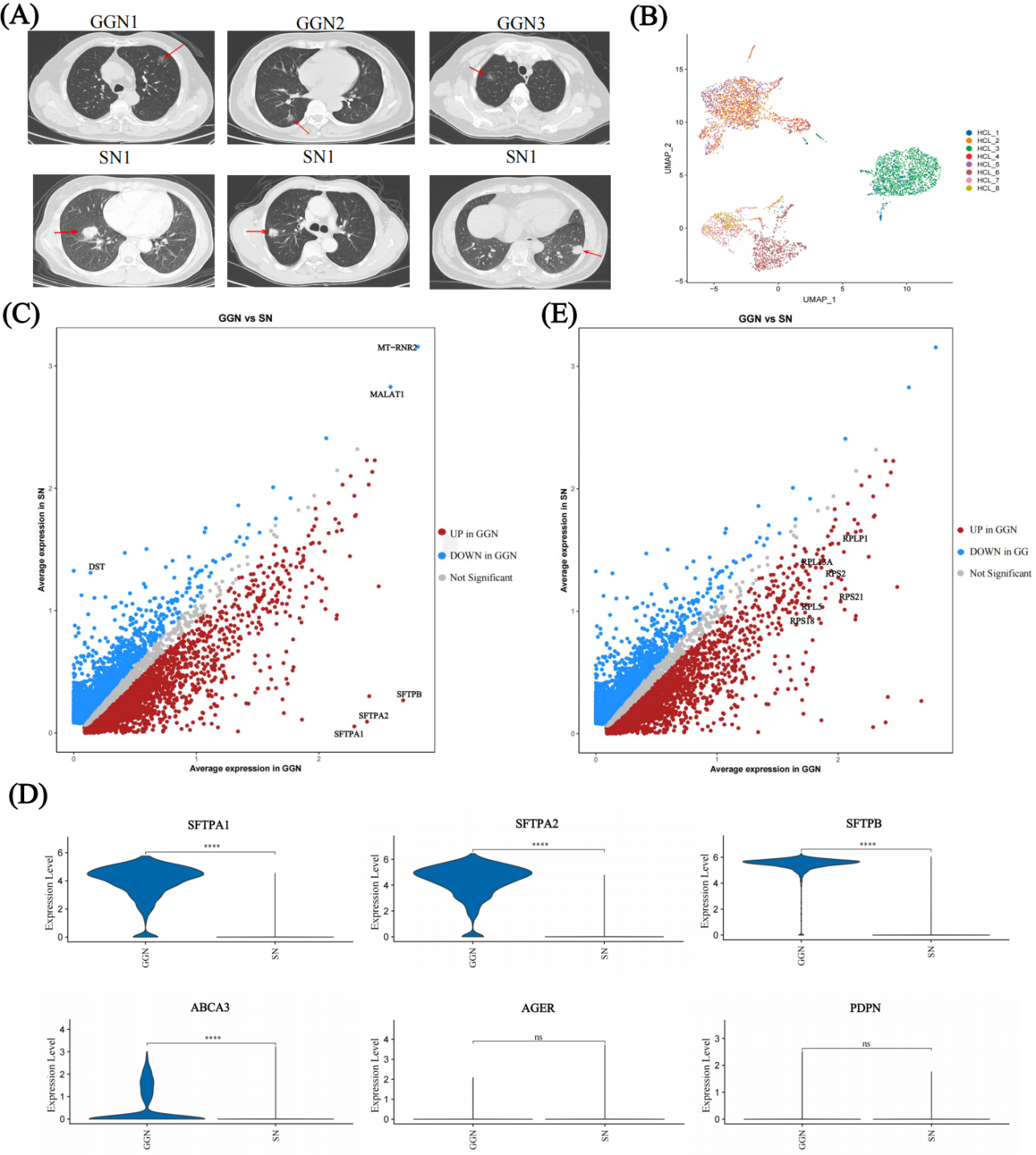
**

**Supplementary Figure 1 (A)**: Radiological features of 3 GGN and 3 SN patients included in this study. Red arrows indicate tumor lesions. **(B)**: UMAP plot showing 8 subclusters of tumor cells according to CNV values.**(C)**:Volcano plot showing the top 3 differentially expressed genes of GGN and SN. The threshold has been set as 0.25 for avg_logFC.**(D)**: Violin plots showing the expression of selected marker genes of cancer cells in GGN and SN. Two-sided unpaired Wilcoxon rank-sum test was used for analysis; all differences with P < 0.05 are indicated, *P < 0.05, **P < 0.01, ***P < 0.001, ns non-significance. **(E)**: Volcano plot showing specific differentially expressed genes of GGN and SN. The threshold has been set as 0.25 for avg_logFC.


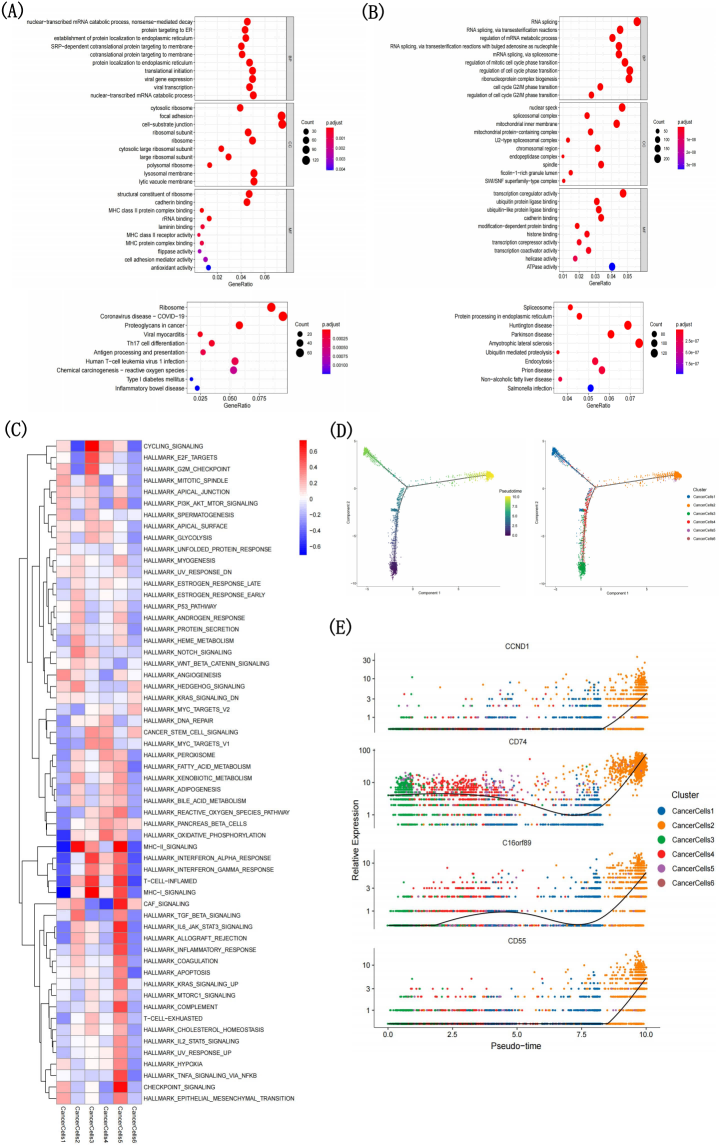


**Supplementary Figure 2 (A, B)**: The bubble plot shows significantly enriched GO and KEGG pathways of cancer cells in GGN**(A)** and SN**(B)**. The color of the bubbles represents the values of significance, and the size represents the number of genes enriched in the pathway. **(C)**: Heat map showing the difference in metabolic pathways scored by GSVA of different cancer cell subclusters. **(D)**: Differentiation trajectory of cancer cells was predicted by monocle 2 **(E)**: Changes in the expression of specific genes in cancer cells with pseudotime. X-axis: Pseudotime change, Y-axis: gene expression.


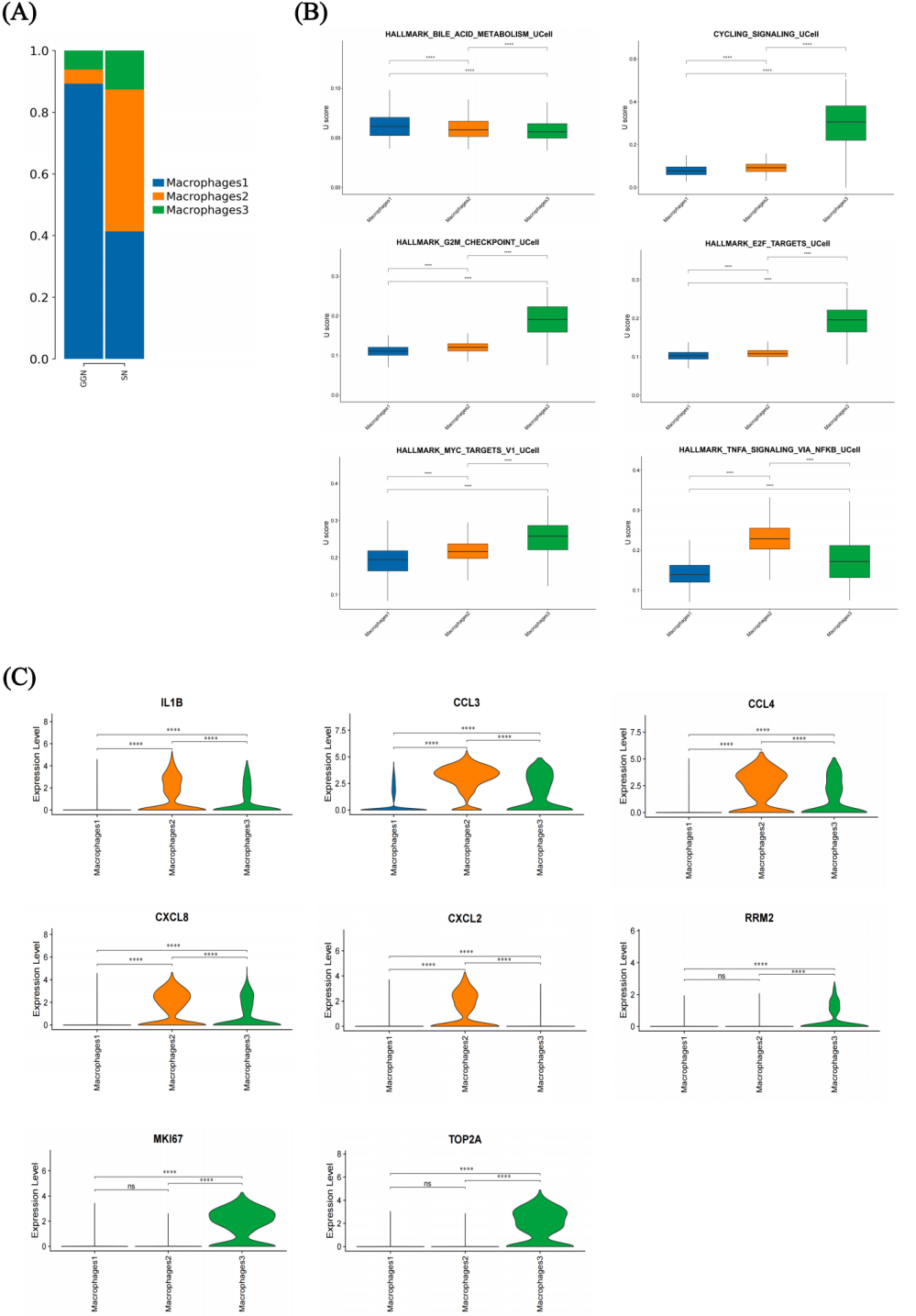


**Supplementary Figure 3 (A)**: Bar plot of the relative percentage of MPS subclusters in GGN and SN.. **(B)**: Box plots of UCell score for different signaling pathways in macrophage subclusters, Two-sided unpaired Wilcoxon rank-sum test was used for analysis; all differences with P < 0.05 are indicated, *P < 0.05, **P < 0.01, ***P < 0.001, ns non-significance. **(C)**:Violin plots showing the expression of selected marker genes of macrophage subclusters. Two-sided unpaired Wilcoxon rank-sum test was used for analysis; all differences with P < 0.05 are indicated, *P < 0.05, **P < 0.01, ***P < 0.001, ns non-significance.


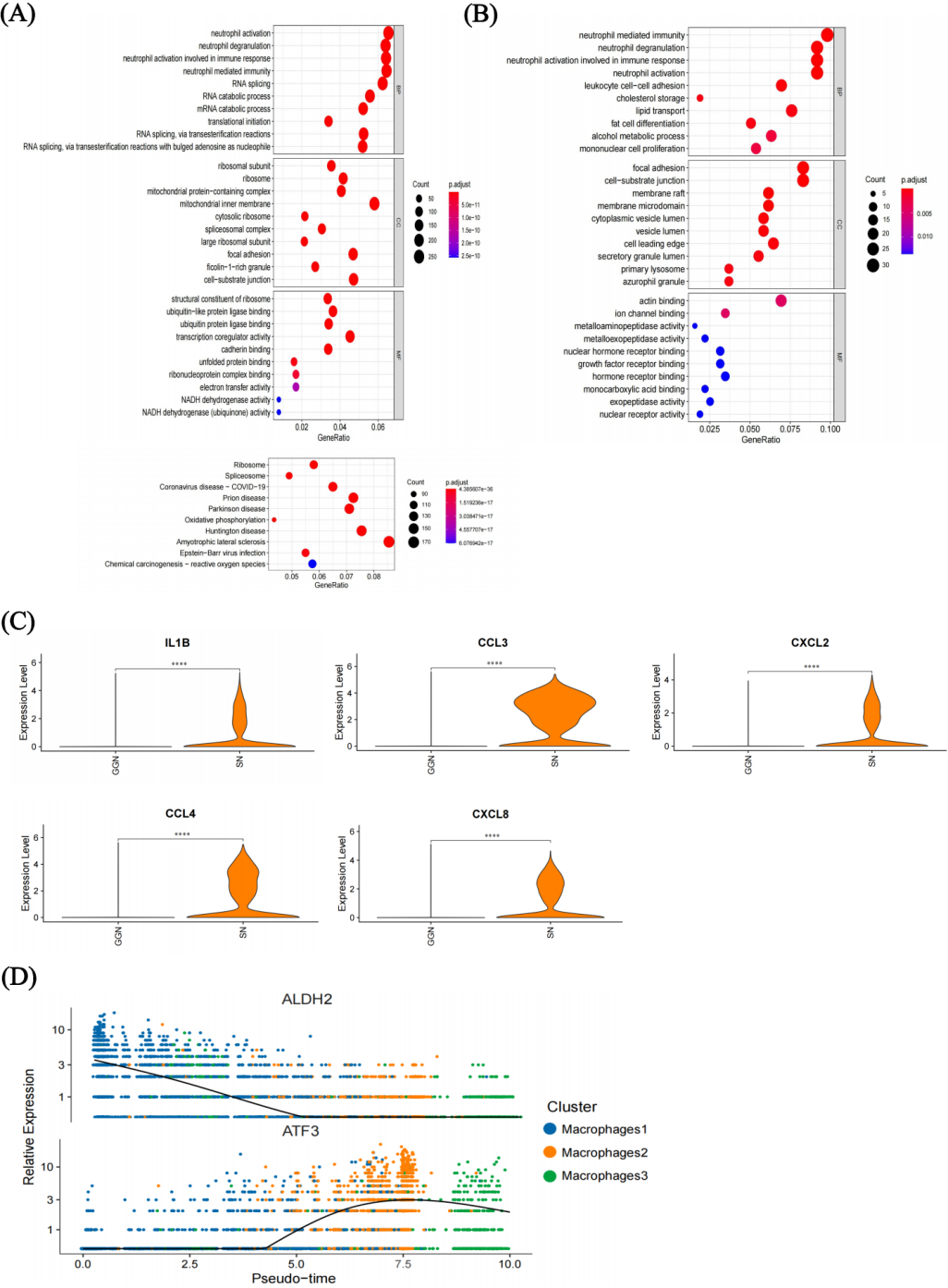


**Supplementary Figure 4 (A)**: The bubble plots show significantly enriched GO and KEGG pathways of macrophages in SN. The color of the bubbles represents the values of significance, and the size represents the number of genes enriched in the pathway. **(B)**: The bubble plot shows significantly enriched GO pathways of macrophages in GGN. The color of the bubbles represents the values of significance, and the size represents the number of genes enriched in the pathway. **(C)**:Violin plots showing the expression of the selected marker genes of macrophages in GGN and SN. Two-sided unpaired Wilcoxon rank-sum test was used for analysis; all differences with P < 0.05 are indicated, *P < 0.05, **P < 0.01, ***P < 0.001, ns non-significance. **(D)**: Changes in the expression of specific genes in macrophages with pseudotime. X-axis: Pseudotime change, Y-axis: gene expression.


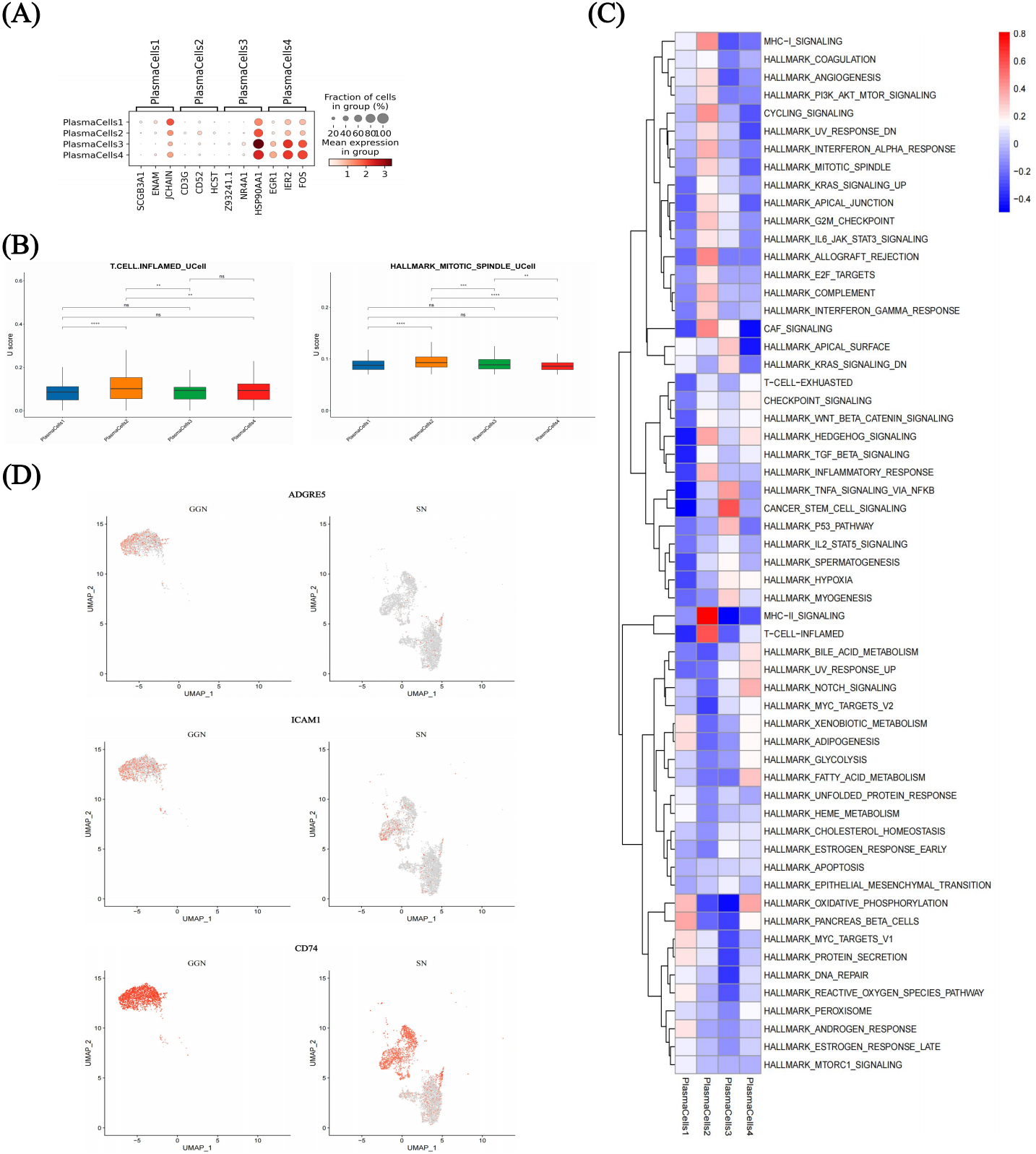


**Supplementary Figure 5(A)**: Bubble plot showing marker genes of plasma cell subclusters. The color of the bubbles represents the average expression level of the gene, and the size represents a fraction of cells. **(B)**: Box plots of UCell score for different signaling pathways in plasma cell subclusters, Two-sided unpaired Wilcoxon rank-sum test was used for analysis; all differences with P < 0.05 are indicated, *P < 0.05, **P < 0.01, ***P < 0.001, ns non-significance. **(C)**:Heat map showing the difference in metabolic pathways scored by GSVA of plasma cell subclusters. **(D)**: The expression of ADGRE5,ICAM1 and CD74 of cancer cells in GGN and SN.


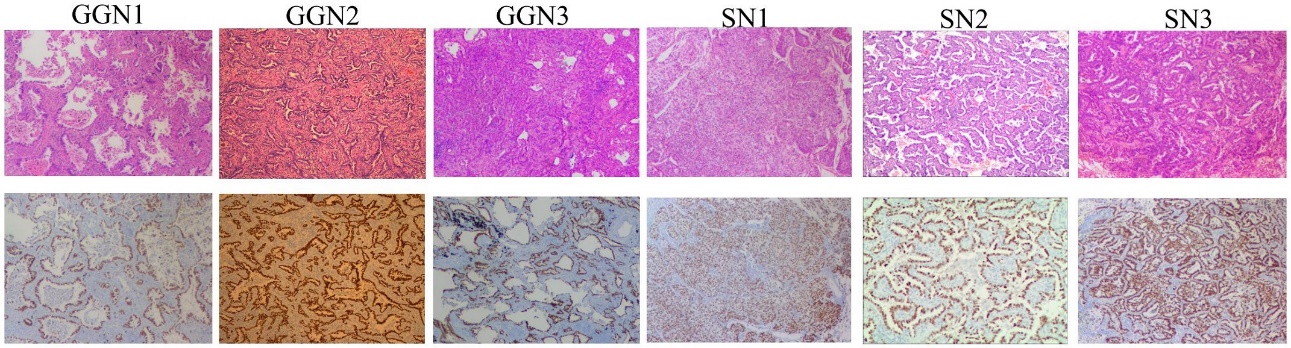


**Supplementary Figure 6** Pathology features containing HE staining (top) and TTF-1 staining (bottom) of six patients.
